# Physico-chemical properties of curcumin nanoparticles and its efficacy against Ehrlich ascites carcinoma

**DOI:** 10.1038/s41598-023-47255-w

**Published:** 2023-11-24

**Authors:** Monira M. Rageh, Eman A. Abdelmoneam, Marwa Sharaky, Ebtesam A. Mohamad

**Affiliations:** 1 Department of Biophysics, Faculty of Science, Cairo University, Giza, Egypt; 2 Pharmacology Unit, Department of Cancer Biology, National Cancer Institute, Cairo University, Cairo, Egypt; 3 Radiology and Medical Imaging Department, College of Applied Medical Science, Prince Sattam Bin Abdul-Aziz University, 11942 Al-Kharj, Saudi Arabia

**Keywords:** Biophysics, Cell biology, Nanoscience and technology

## Abstract

Curcumin is a bioactive component with anticancer characteristics; nevertheless, it has poor solubility and fast metabolism, resulting in low bioavailability and so restricting its application. Curcumin loaded in nano emulsions (Cur-NE) was developed to improve water solubility and eliminate all the limitations of curcumin. Size distribution, zeta potential, transmission electron microscopy (TEM) measurements, UV–Visible spectra, IR spectra and thermogravimetric analysis (TGA), were used to characterize the prepared Cur-NE. Cancer therapeutic efficacy was assessed by oxidative stress (superoxide dismutase (SOD), Glutathione–S–Transferase (GST), malondialdehyde (MDA) and nitric oxide (NO), DNA damage, apoptotic proteins (caspase-3 and 9), besides investigating tumor histology and monitoring tumor growth. Additionally, the cytotoxicity and genotoxicity of the liver, kidney, heart, and spleen tissues were examined to gauge the adverse effects of the treatment method’s toxicity. The results showed that Cur-NE is more effective than free curcumin at slowing the growth of Ehrlich tumors while significantly increasing the levels of apoptotic proteins. On the other hand, Cur-NE-treated mice showed some damage in other organs when compared to mice treated with free curcumin. Cur-NE has a higher efficacy in treating Ehrlich tumor.

## Introduction

Cancer has become one of the most serious health issues in our decade, according to the American Cancer Society. Cancer is a term used to describe a group of cells that have oddly spread, invasion and metastasis^1,2^, and the most common reason for mortality is cancer in humans after cardiovascular disorders. Although great efforts have been made in developing treatment regimens such as chemotherapy, surgery, and radiotherapy, they have a significant negative impact on health. Therefore, several studies have been devoted to researching new methods aimed at finding treatments that have anti-proliferative effects on cancer cells while having less immune system side effects. As a result, researchers are investigating the combination between nanoscale particles and natural components (plants, animals, and microorganisms), which are considered novel strategies for cancer treatment^3–6^.

Natural phytochemicals have received several interests as anticancer and ant-inflammation medicines since they are less toxic to normal cells and have less side effects when compared to chemical medications^7^. The metabolites of phytochemicals, which also include alkaloids, flavonoids, phenolics, tannins, glycosides, gums, resins, and oils, have high anticancer potential and serve a variety of therapeutic purposes in human systems. There are various methods by which phytochemicals have anticancer effects. They selectively kill rapidly dividing cells, target incorrectly expressed molecular factors, eliminate oxidative stress, alter cell growth factors, block angiogenesis of malignant tissue and cause apoptosis^8,9^.

Curcumin is a natural hydrophobic polyphenol derived from the *Curcuma longa* plant that is suspected to have anticancer, chemo preventive, anti-inflammatory^10,11^, antibacterial, and antioxidant effects. Because of its several methods of action, it has a wide spectrum of qualities^12–14^. Curcumin’s anticancer properties are mediated through several methods. Curcumin triggers oxidative reactions and mitochondrial membrane breakdown by increasing ROS generation. As a result, the cell cycle is disrupted, and apoptosis is triggered. Curcumin interacts with enzymes, transcription factors, members of the Bcl-2 family, death receptors, and apoptotic proteins. As a result, cell proliferation is slowed^15–17^.

Unfortunately, all of curcumin’s beneficial chemoprotective effects are limited due to several major issues, including poor bioavailability, low solubility in water, rapid excretion, and higher rate of metabolic activity^18^. As a result, numerous technological methods for overcoming these issues and improving their qualities have been investigated. Wang et al.^19^ used full encapsulation of curcumin in sodium alginate/ZnO hydrogel beads to modulate curcumin delivery. Suryani et al.^20^ used low viscosity chitosan and tripolyphosphate at various concentrations to make curcumin nanoparticles, which they recommended as a promising anticancer treatment. Elbialy et al.^21^ produced iron oxide nanoparticles coated with curcumin to improve curcumin circulation and biodistribution. Also, Elbialy et al.^22^ encapsulated curcumin in mesoporous silica nanoparticles and reported that the formulation was useful for cancer therapy. Ben et al.^23^ employed a microemulsion to encapsulate the curcumin and used it as radioprotective.

In the current study, it is critical to investigate the synthesis and characterization of curcumin encapsulated in nano-emulsions, as well as its release. Secondly, the antitumor protentional of curcumin nano-emulsions compared with native curcumin in the in vivo system using Ehrlich tumor bearing mice as a model. Thirdly, the side effects on the vital organs (kidney, heart, spleen, and liver) were evaluated for all the treated mice groups and compared with untreated mice.

## Materials and methods

### Materials

Curcumin, lauric acid, Span® 20 (sorbitan laurate), Tween® 80 (polysorbate 80) and isopropyl myristate were acquired from Sigma-Aldrich and utilized without extra refining. Phosphate buffer (PBS) was obtained from MP Biomedical, LLC (USA), and nitric oxide assay (NO), Glutathione–S–transferase assay, lipid peroxidation (MDA) and superoxide dismutase (SOD) kits were obtained from bio diagnostic Co., Giza, Egypt.

### Preparation of curcumin loaded in nano emulsions (Cur-NE)

Curcumin loaded in nano emulsion (Cur-NE) was created using the techniques described in Ben et al. and Kesisoglou and Panmai^23–25^. In a nutshell, Span® 20 (0.16 mL), Tween® 80 (3 mL), isopropyl myristate (0.6 mL), and lauric acid (100 mg) were mixed until a clear liquid was formed. Curcumin (125 mg) was added to the solution and stirred with a magnetic stirrer (MS-300HS, Misung Scientific, Gyeonggi-do, Korea) until the components were completely combined before adding 8 mL of distilled water drop by drop. It may take up to 24 h for curcumin nanoparticles to equilibrate. At room temperature, all processes were accomplished.

### Characterization of curcumin free (CR) and curcumin nano emulsions (Cur-NE)

The size and shape of Cur-NE were observed by Transmission Electron Microscope (TEM, FEI Tecnai G20, Super twin, Double tilt, LaB6 Gun). The mean particle size, size distribution, and zeta potential for Cur-NE were examined by the dynamic light scattering device (Malvern Zetasizer Nano ZS Instruments Ltd, UK). Additionally, the structural characteristics of CR and Cur-NE were determined by the absorption spectra (JENWAY, 6405 UV/VIS. Spectrophotometer, Barloworld scientific, Essex, UK, λ = 250–600 nm), Fourier-Transform Infrared Spectroscopy (Basic Vector, 22FT-IR, Germany, λ^−1^ = 4000 and 400 cm^−1^ with 4 cm^−1^ resolve) and thermogravimetric analyzer (TGA) (DTG-60H, SHIMADZU, Japan, temperature range room temperature up to 400 °C at a rate of 10 °C/min). Furthermore, the encapsulation efficiency of curcumin in a nano emulsion and its release was performed accordingly to Avgoustakis et al.^26^, briefly, first, the encapsulation efficiency of Cur-NE was performed by measuring the absorbance of curcumin at different concentrations using a UV–Visible spectrophotometer at 435 nm. The standard curve of curcumin was prepared by plotting optical density against the concentrations and same was used for determining the concentration of curcumin in the supernatant. Second, the release of curcumin from Cur-NE was performed as followed, two suspensions of Cur-NE and CR were in two cellulose acetate dialysis chambers (Spectra/P or. MW cut-off 12,000, Spectrum, Canada), dipped in buffer (phosphate solution: Tween 8:2) and stirred at 50 rpm. At the waiting times, 2 mL of release buffer was withdrawn and immediately an extra 2 mL of new buffer was added and kept for up to 12 h. Curcumin concentrations in the release buffer were measured by spectrophotometry at 430 nm.

### Animals and experimental design

**Figure 1:**
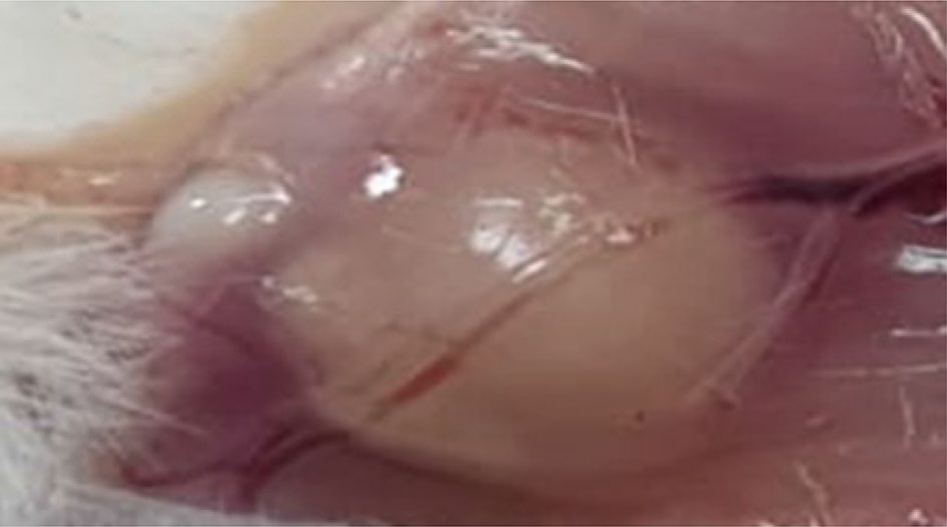
Image of tumor formed in the mice.

Based on a review of approval number CU/I/F/1/21, the Cairo University Research Ethics Committee granted approval for a total of 20 albino mouse procedures and experimental protocols that were carried out in accordance with the Guide for the Care and Use of Laboratory Animals and Use Committee (CU-IACUC), which provide the policies, procedures, standards, organizational structure, staffing, facilities, and practices to achieve the humane care and use of animals in the laboratory. The National Cancer Institute (“NCI”), Cairo University, Giza, Egypt, provided male albino mice (average weight 25 g, 8–10 weeks old). The mice were housed in the lab for a week before the test as acclimatization under standard laboratory conditions. Separate cages were used to house the mice, and they had unrestricted access to clean, fresh water. Ehrlich ascites carcinoma (EAC) (Ehrlich ascites mammary carcinoma is a spontaneous murine mammary adenocarcinoma and it is one of the well-established models in cancer biology^27^ was bought from Cairo University’s Tumor Biology Department of the National Cancer Institute (“NCI”). The experimental production of solid tumor in male mice was carried out by subcutaneous injection of 0.1 mL of Ehrlich’s ascites carcinoma (2.5 × 10^6^ cells/mL) to the right hind limb of each mouse under sterile conditions. 10–13 days after tumor injection, the tumor manifested and became palpable in the injected animals as shown in Fig. 1 and described by Abdelrahman et al.^28^.

**Figure 2:**
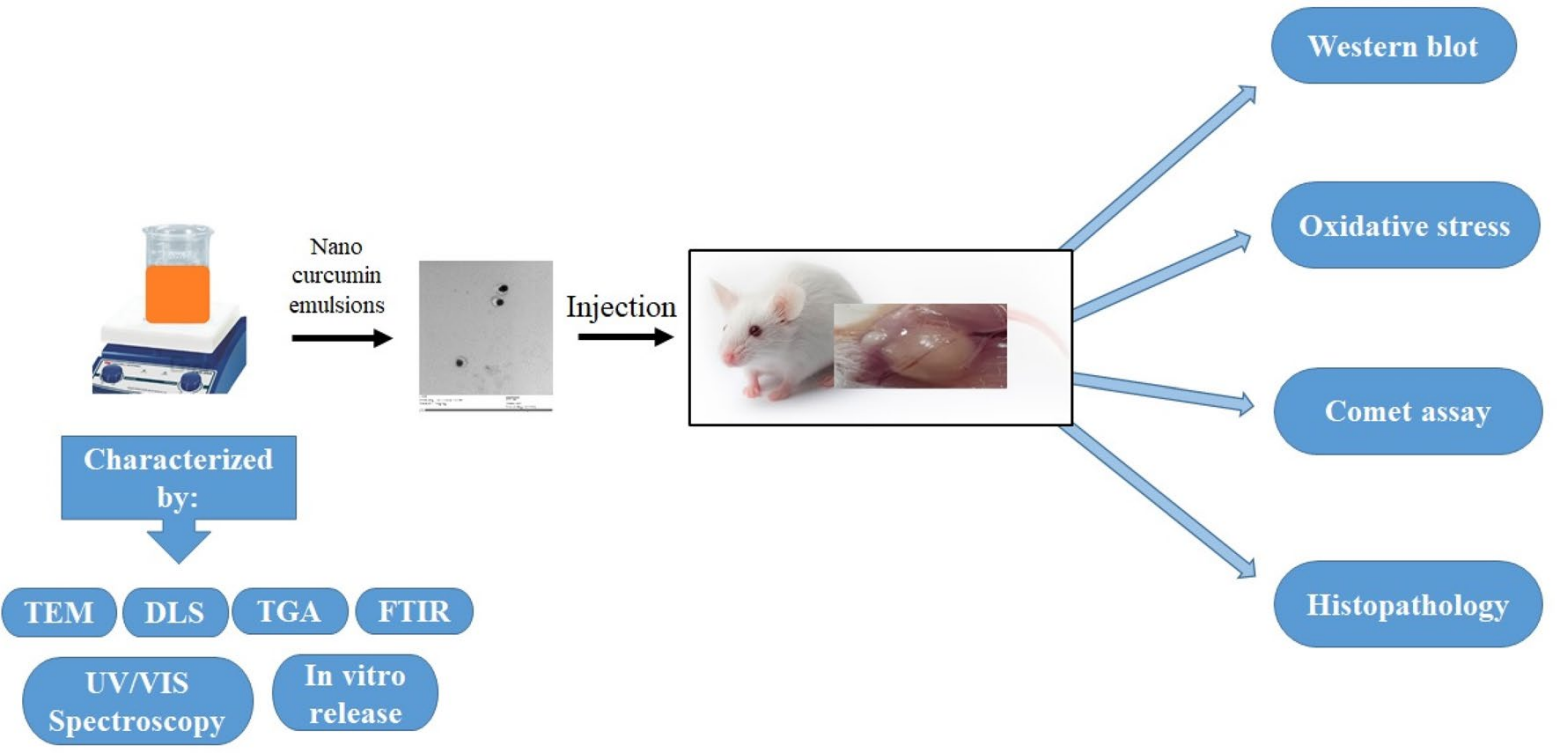
Shows a graphical illustration of the experimental techniques, as well as the study design and evaluation parameters.

Mice were separated into four groups (n = 5) at random: control, tumor, CR and Cur-NE group. Control and tumor groups were received orally PBS, CR and Cur-NE groups were received orally 20 mg/kg curcumin extract and 20 mg/kg curcumin loaded in nano emulsions respectively, every day for 2 weeks. Through the treatment procedure, the tumor size was examined every 3 days across a period of 12 days for all the experimental groups and estimated based on Ghous et al.^29^. At the end of the treatment protocol, all mice were sacrificed by sudden decapitation; tumor tissues, kidney, heart, spleen, and liver were instantly removed from each, cleaned with isotonic saline, split, and used for assessment (Fig. 2).

### Oxidative stress analysis

The tumor, kidney, heart, spleen, and liver tissues of all mice groups were homogenized in cold phosphate-buffer, centrifuged at 4000 rpm (VS18000 M; Vision scientific, Korea) for 10 min and the supernatants were used for measuring the oxidative stress. The activity of superoxide dismutase (SOD) and Glutathione–S–Transferase (GST) and the level of malondialdehyde (MDA) and nitric oxide (NO) were determined using superoxide dismutase assay kit (SOD 2521), the Glutathione–S–Transferase assay kit (GST 2519), lipid peroxidation assay kit (MDA 2529), and nitric oxide assay kit (NO 2533) respectively, according to the manufacturer’s instructions.

### Comet assay

Comet assay is a quick, easy, visible, and exact way to assess DNA destruction and the early stages of apoptosis^30^. DNA damage induced in tumor, kidney, heart, spleen, and liver tissues for all experimental groups was estimated by the Comet assay described by Rageh and El-Gebaly^31^. Briefly, comet assay was done under alkaline conditions (pH > 13), a sample (5 μL) of cell suspension was mixed with70 μL of 0.7% low-melting-point (LMP) agarose. Agarose was put on a microscope slide, which was previously covered with a thin layer of 0.5% normal melting point (NMP) agarose. After cooling at 4 °C for 5 min, slides were wrapped with a third layer of LMP agarose. After solidification at 4 °C for 5 min, slides were immersed in recently prepared cold lysis solution at 4 °C for at least 1 h. Subsequent lysis, slides were put in a horizontal gel electrophoresis unit and sit on in fresh alkaline electrophoresis buffer. Electrophoresis was done for 30 min at 24 V (∼ 0.74 V/cm) and 300 mA at 4 °C. Therefore, the slides were dipped in neutralizing buffer and lightly washed times for 5 min at 4 °C. All the procedures were completed under lowered light. Comets were visualized by ethidium bromide staining solution and examined at × 400 magnification using a fluorescence microscope. Comet 5 image analysis software exploited by Kinetic Imaging, Ltd. (Liverpool, UK) linked to a CCD camera was used to assess DNA damage in the cells by measuring the length of DNA migration and the percentage of migrated DNA. Tail moment and Olive moment were computed^32^.

### Western blots

Western blotting analysis of caspase-3 and caspase-9 was completed using the procedure outlined in Lihua et al.^33^. All mice groups’ tumor tissues were homogenized, centrifuged and lysed in the radio-immunoprecipitation assay (RIPA) lysis buffer including a protease/phosphatase inhibitor cocktail and placing them on ice for 30 min until complete lysis was achieved. The lysate was transferred to an Eppendorf tube and centrifuged at 4 °C for 15 min at 13,000 rpm (VS18000 M; Vision scientific, Korea). SDS-PAGE (12% acrylamide) was used to separate the extracted proteins, which were then stained onto PVDF membranes. For normalization, antibodies against-actin were used to probe the membranes. The target proteins’ band intensities were compared to the control sample-actin (housekeeping protein) using protein normalization on the ChemiDoc MP imager using image analysis software.

### Histological examination

All groups’ tumor samples were embedded in paraffin bars, fixed in 10% neutral buffered formalin solution, sectioned into 5 µm thick sections, and stained with hematoxylin and eosin (H&E) as pathologists and researchers usually utilize this stain as an initial evaluation since it gives a thorough picture of the microanatomy of a tissue^34^. For the inspection of tissue slices, an optical microscope (CX31 Olympus microscope, Tokyo, Japan) connected to a digital camera (Canon) was employed.

### Statistical analysis

The data was examined using SPSS 19.0 for Windows, and the mean and standard deviation (SD) were displayed. One-way analysis of variance (one-way ANOVA) was used to calculate significant differences between groups; significant difference (LSD) was applied to multi-group evaluations. It was deemed significant at P ≤ 0.05.

## Results and discussion

Curcumin is a polyphenol chemical found in nature. It’s regarded as a powerful anti-inflammatory and antioxidant. Because of these characteristics, it can be used to treat a wide range of cancers. Curcumin’s clinical use has been limited due to its lack of solubility, stability, bioavailability, and selectivity. As a result, several initiatives have been made in recent years to eliminate these problems and improve clinical applications, such as nano carriers, nanoparticles, micelles, solid dispersions and liposomes. The present study highlights the use of curcumin loaded in nano emulsions (Cur-NE) which has shown several major advantages, including higher physical stability and drug loading ability, low toxicity, and controlled drug release, and targeting^18,35–39^.

### Cur-NE characterization

**Figure 3:**
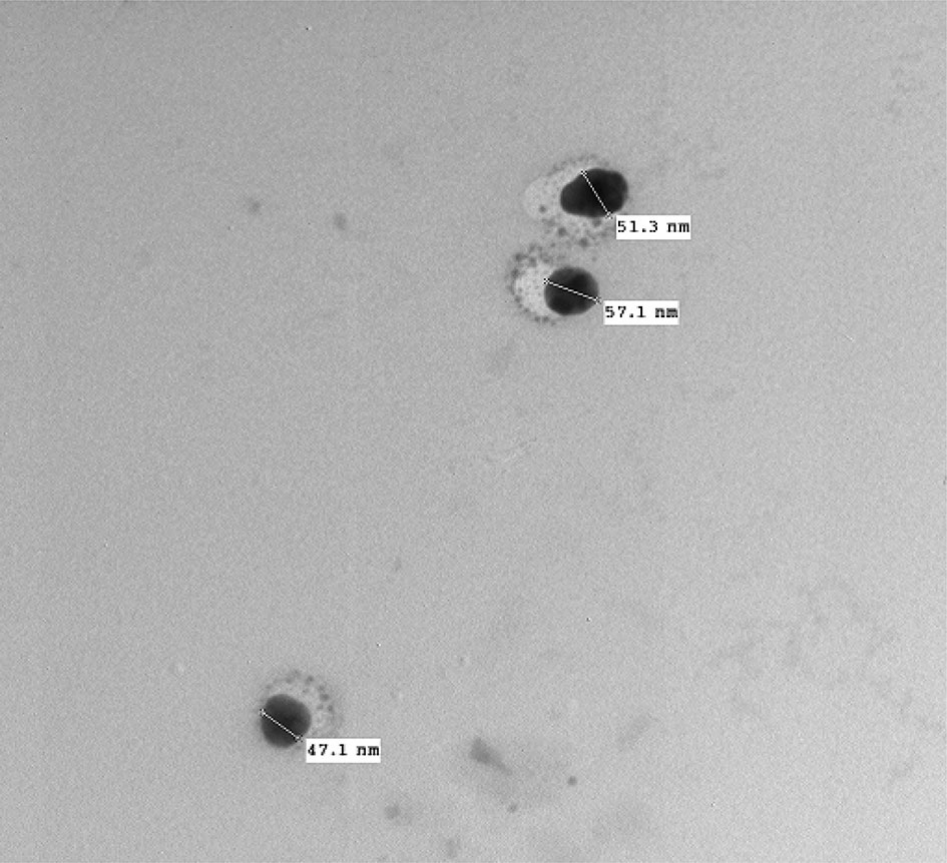
Transmission electron microscope (TEM) image of curcumin loaded in nano emulsions (Cur-NE).

**Figure 4:**
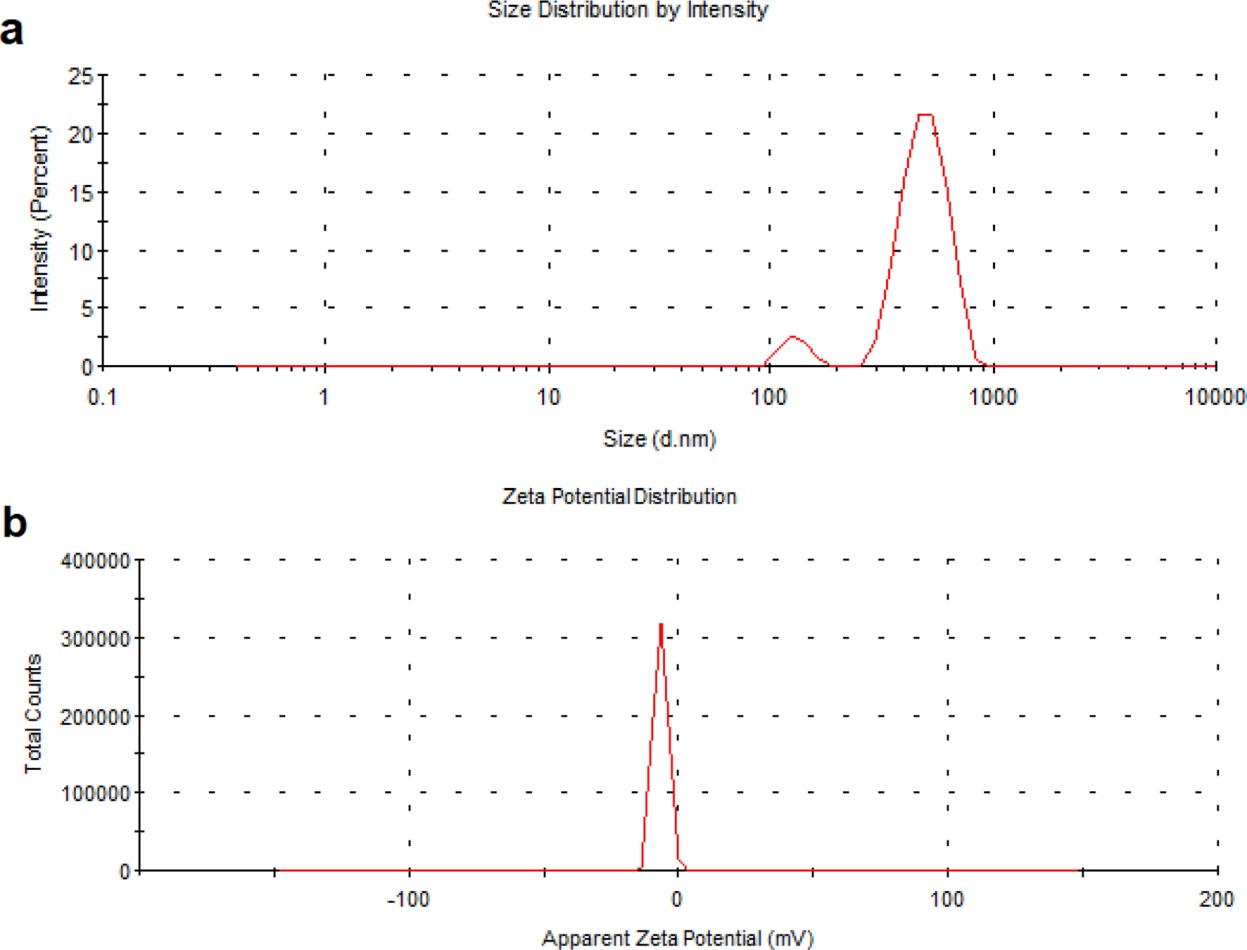
Size distribution (**a**) and zeta potential (**b**) of curcumin loaded in nano emulsions (Cur-NE) measured by dynamic light scattering. The data points are the means of three independent measurements.

In the present study, Cur-NE was set according to the method of Ben et al. and Kesisoglou and Panmai^23–25^ and characterized using several techniques. The shape and size of Cur-NE were observed by TEM (Fig. 3). The round nanoparticles in the TEM image are evenly distributed, not aggregated, and range in diameter from 45 to 60 nm. The dynamic light scattering (DLS) measurement shown in Fig. 4a supports this finding. The size of Cur-NE is centered at 459 nm, (PDI = 0.604) with a reasonably narrow distribution but appears to be higher compared to TEM due to the interference of the dispersant to the hydrodynamic diameter. The potential analyzer was used to determine the electro-kinetic surface potential for Cur-NE (Fig. 4b). The value of zeta potential for Cur-NE is (− 16 mV ± 1.2). This result revealed that the particles were stable and evenly spread. These results matched those of Paolino et al.^40^, who stated that particles with a high negative or positive zeta potential are suspended. Because the particles are opposing each other, there will be no desire for them to stick together.

**Figure 5:**
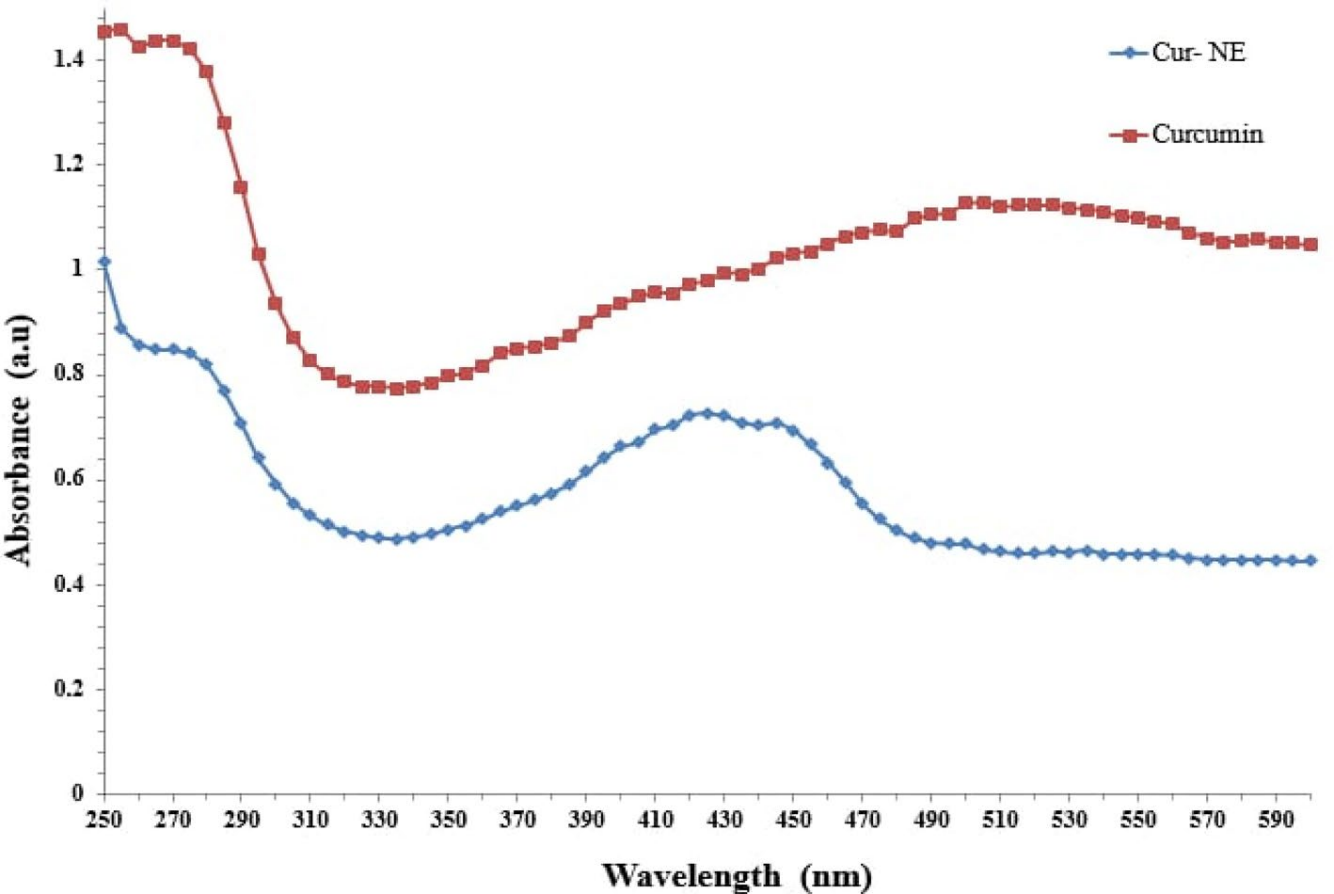
UV–Visible spectra of curcumin free (CR) and loaded in nano emulsions (Cur-NE).

UV–Vis and FT-IR spectra were employed to support curcumin free (CR) and Cur-NE structure. Figure 5 shows the absorption spectra of CR and Cur-NE in the wavelength range 250–600 nm, where the absorption peaks centered at 510 nm and 420 nm for CR and Cur-NE respectively. The shift between the CR and Cur-NE absorption bands might be attributed to Cur-NE production and the alterations in the structure or chemical environment of the Cur-NE this result is consistent with Pandit et al. and Kumar et al.^41,42^.

**Figure 6:**
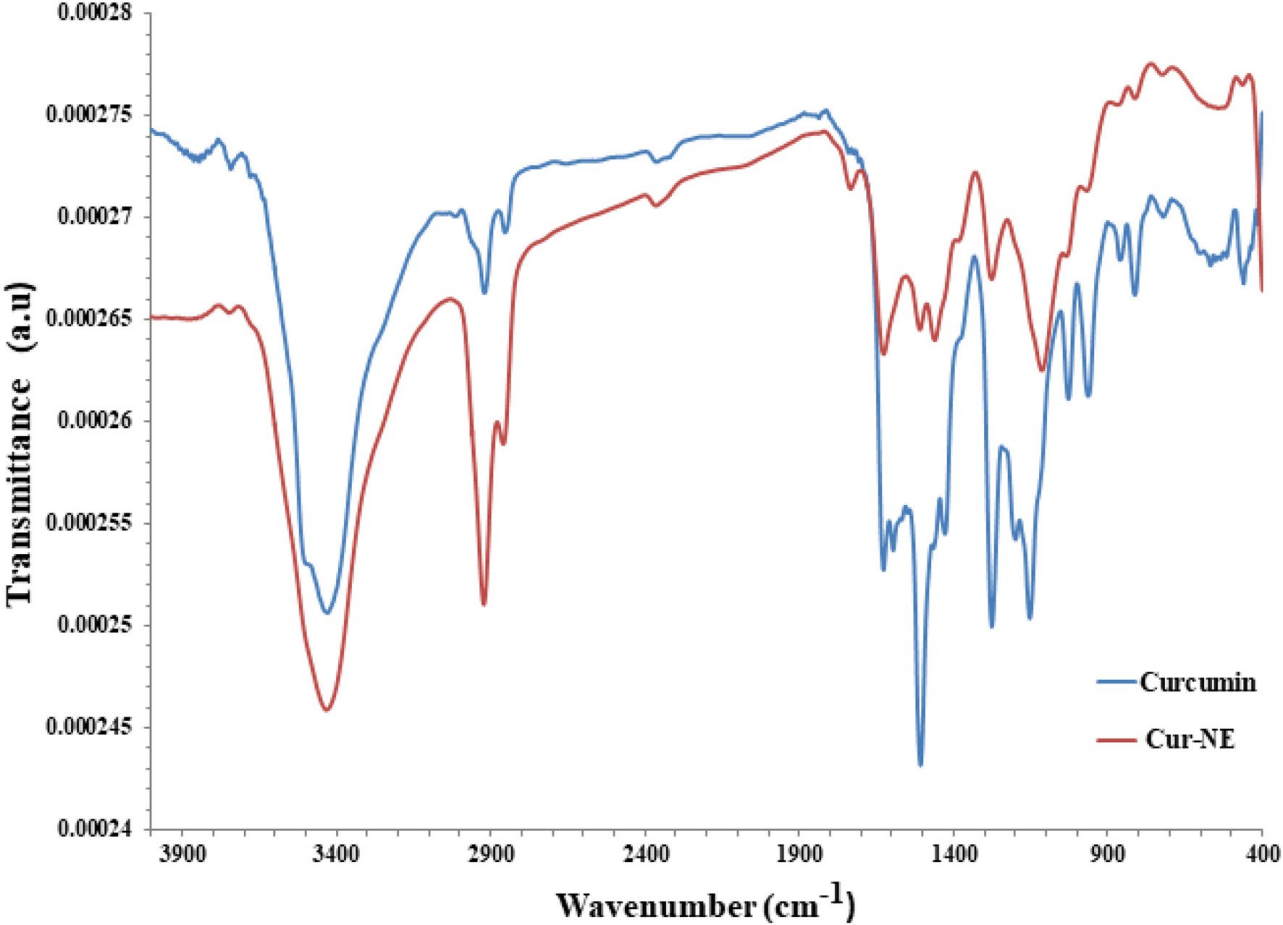
FT-IR spectra of curcumin free (CR) and loaded in nano emulsions (Cur-NE).

The FT-IR spectra of CR and Cur-NE are presented in Fig. 6. Both samples exhibited characteristic peaks around 3532 cm^−1^ (O–H), 1500 cm^−1^ (–C=O), 1424 cm^−1^ (C–H), 1025 cm^−1^ (C–N) to 3450 cm^−1^ (O–H), 1465 cm^−1^ (–C=O), 1100 cm^−1^ (C–H), 960 cm^−1^ (C–N), 1624 cm^−1^ (C=C), 1591 cm^−1^ (benzene ring stretching vibrations), 1272 cm^−1^ (C–O), 1024 cm^−1^ (C–O–C)^43,44^. The spectra of CR and Cur-NE indicates a few changes in the structure which happened at the molecular level during the preparation of the nanocurcumin^45^.

**Figure 7:**
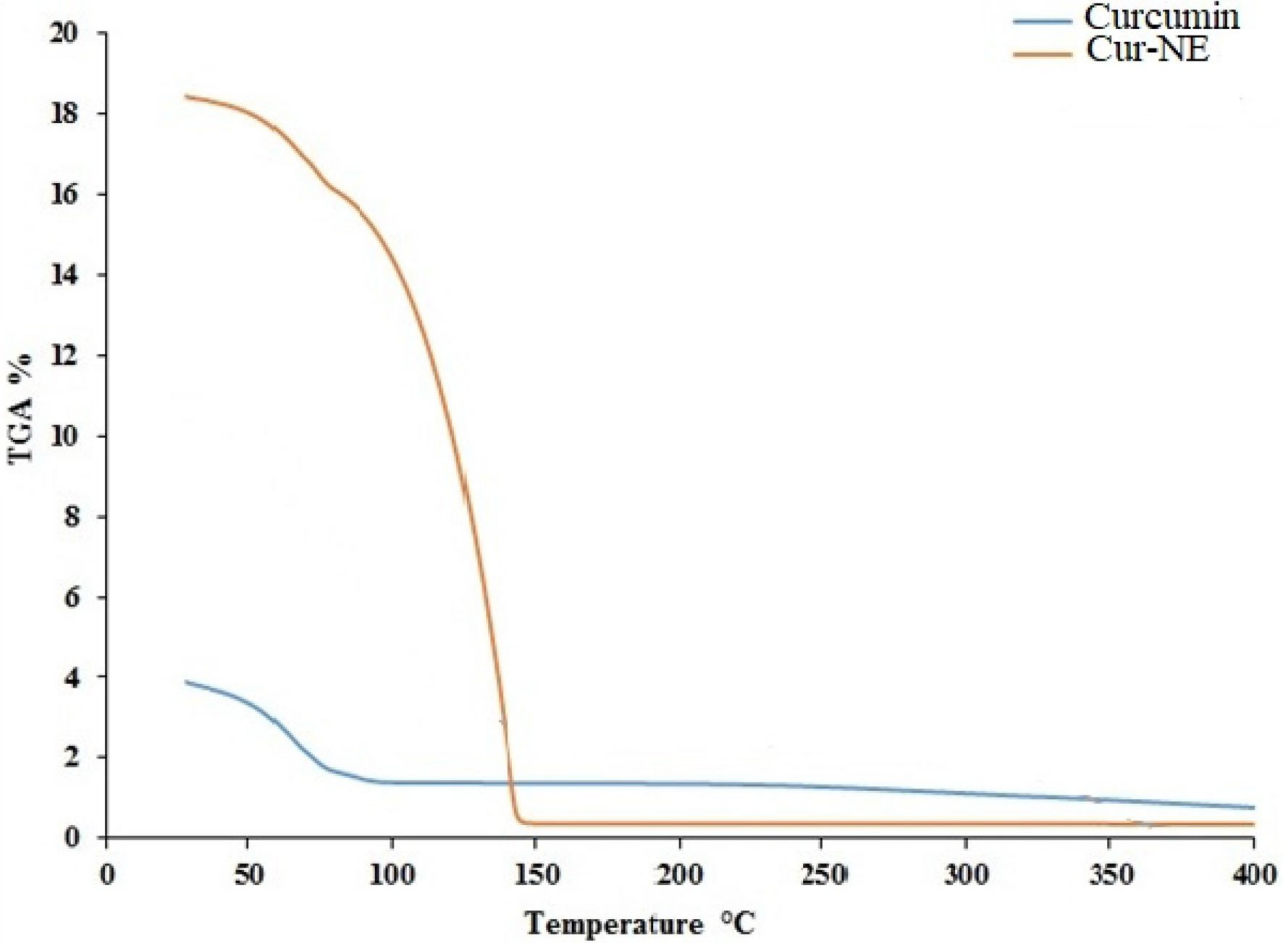
Thermogravimetric analysis (TGA) of curcumin free (CR), loaded in nano emulsions (Cur-NE).

Thermal analyses are necessary to determine the thermal variations in compounds and the maximum temperature to which they can be exposed without losing their properties. Thus, thermogravimetric analysis (TGA), which tracks mass variations, was used to assess CR and Cur-NE thermal stability (Fig. 7). The TGA curves of CR and Cur-NE indicated to a weight loss 2% and 18% in temperature ranges 25–75 °C and 25–150 °C respectively, the increase in weight loss in Cur-NE might be attributed to the present of water and organic solvents in the formulation. Moreover, higher decomposition temperature suggests that increased thermal stability and heat tolerance for Cur-NE^46,47^. Furthermore, encapsulation efficiency of loaded curcumin in nano emulsions (NE) is an important characteristic, hence, at the current study, the encapsulation efficiency of curcumin in Cur-NE was 85%. This finding suggests that the NE may keep a high quantity of curcumin^48^ due to stronger non-covalent interactions between the curcumin and the compounds of the formulation and/or due to the bigger volume available to incorporate the curcumin.

**Figure 8:**
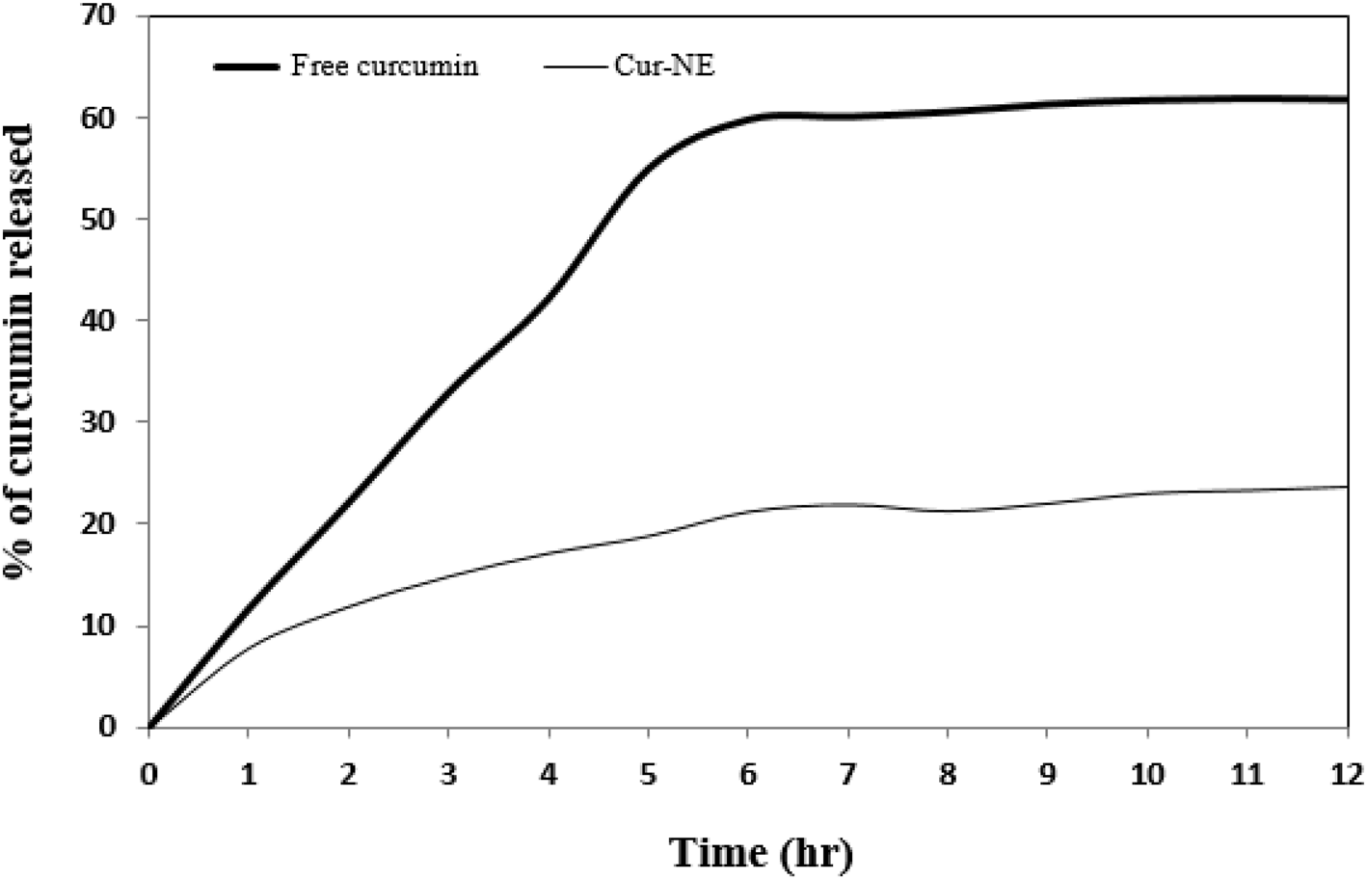
In vitro release of curcumin free (CR) and loaded in nano emulsions (Cur-NE).

Figure 8 reveals the performance of in vitro release of free curcumin and Cur-NE. As shown in the figure, more than 60% of the free curcumin is released within 6 h, while Cur-NE displays gradual release around a long period of time with just 20% curcumin released within 6 h. The in vitro release study of curcumin displayed no outbreak result so that the drug passage out of the nanoparticles was regulated mainly by a diffusion-controlled mechanism. Mohamad et al.^49^ reported that slow release due to intermolecular interaction with the near environment. Furthermore, the NE is suitable for drug carriers due to high encapsulation efficiency and control the release. Hossann et al.^50^ confirmed this finding, reporting that the shielding effect of the lipid bilayer in liposomes mediated drug delivery was observed to be sustained and prolonged drug release pattern to the targeted areas.

### Cur-NE therapeutic efficacy in solid Ehrlich carcinoma-bearing mice

Tumor size, oxidative stress, histology, DNA damage and apoptotic proteins were used to assess Cur-NE therapeutic efficacy in solid Ehrlich carcinoma-bearing mice. Oxidative stress and DNA damage were also considered in the liver, kidney, heart, and spleen to recommend the suggested treatment mode, where these organs are the initial sites for the accumulation of foreign substances associated with the pathogenesis of systemic diseases^51^.

### Oxidative stress

**Figure 9:**
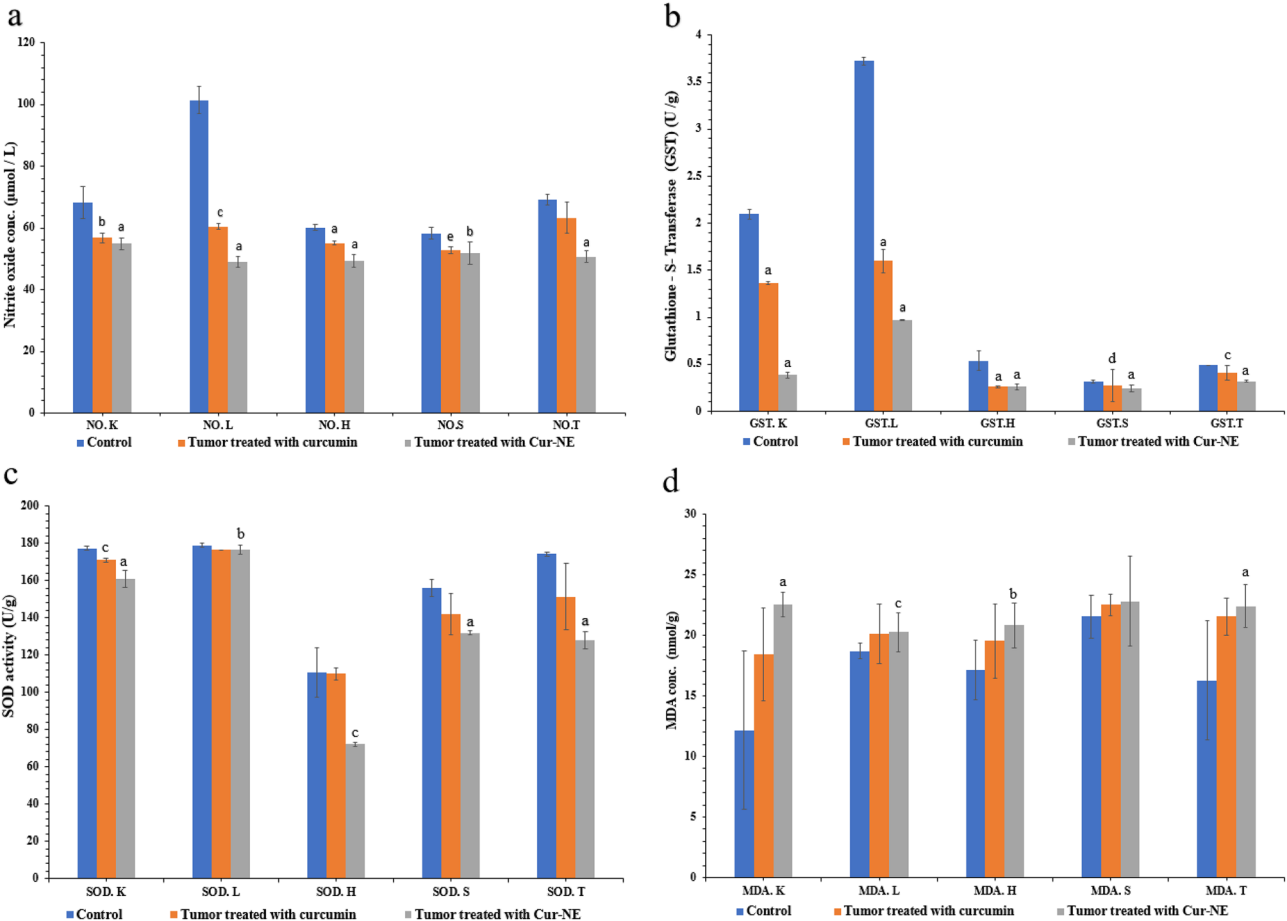
NO level (**a**), GST activity (**b**), SOD activity (**c**) and MDA level (**d**) in kidney (K), liver (L), heart (H), spleen (S), and Ehrlich tumor (T) excised from all experimental groups. The data points are represented as mean ± SD (n = 3). Statistical difference denotes at a, p ≤ 0.0001, b, p ≤ 0.001 and c, p ≤ 0.002 compared to control group.

When compared to normal cells, cancer cells produce reactive oxygen species (ROS) at a higher rate, altering the redox environment. MDA is a biomarker of oxidative stress since it is the byproduct of lipid peroxidation, and its level is associated with tumor progression. Simultaneously, SOD activity is lowered to protect the animals from ROS^52–54^. Moreover, most chemotherapeutics mediators raise intracellular levels of ROS, and can alter redox homeostasis of cells^55^. Consequently, treatment with curcumin free and Cur-NE were evaluated by the activity of SOD, GST and the level of MDA and NO in kidney, liver, heart, spleen, and tumor tissues (Fig. 9a–d). Figure 9a demonstrates a significant decrease in NO levels in the range (20–50%), in the mice group treated by Cur-NE compared to control group. Figure 9b,c, show a significant decrease in the activity of GST and SOD in the range (35–80%) and (2–35%) respectively in the mice group treated by Cur-NE compared to the control one. Also, Fig. 9d shows a significant increase in the level of MDA in the range (40–85%) in the mice group treated by Cur-NE compared to the control one. These results are coherent with several studies that documented curcumin treatment combined with increased ROS production and disruption of the antioxidant system^56–59^.

### Comet assay

**Figure 10:**
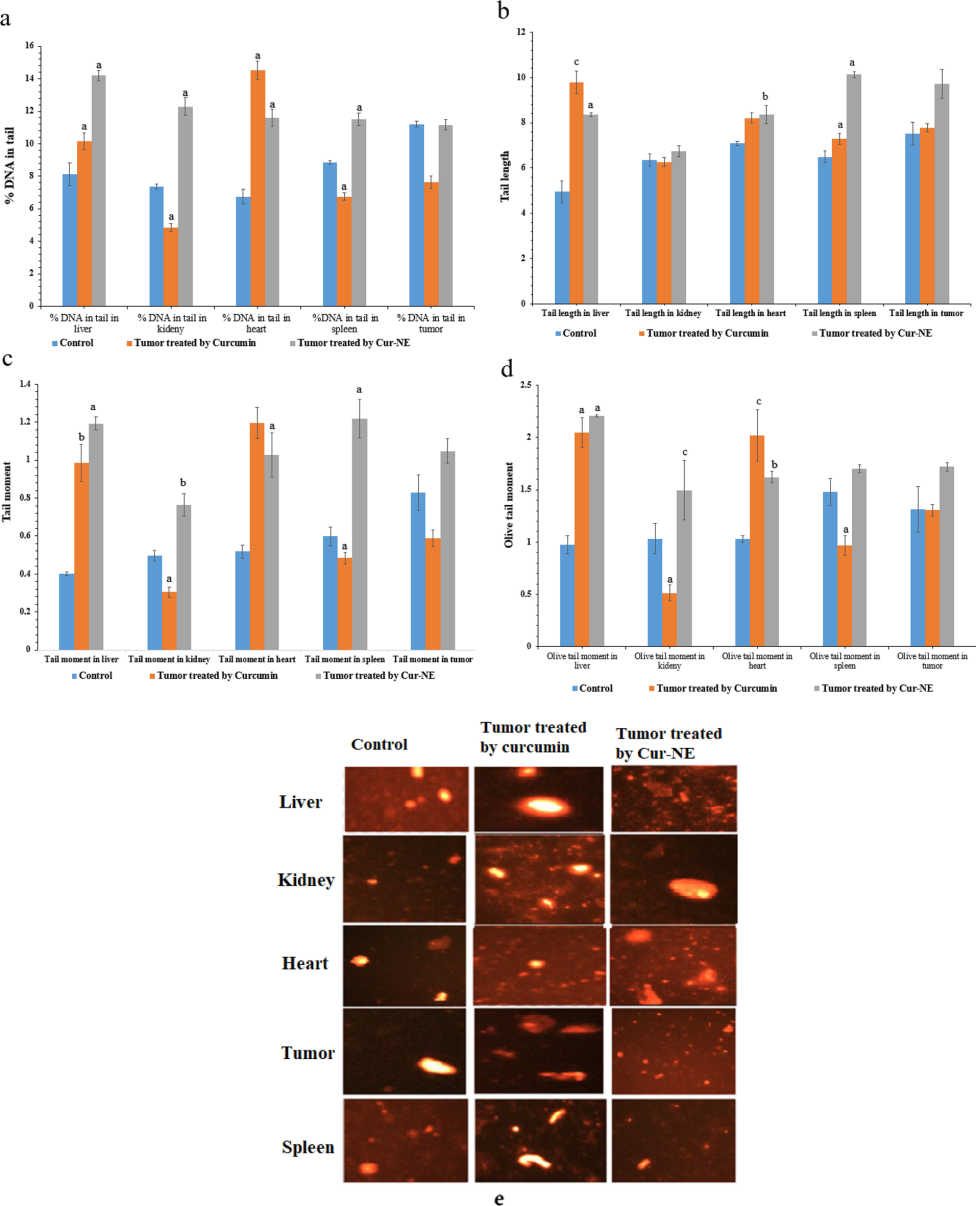
Comet parameters of DNA from kidney, liver, heart, spleen, and tumor tissues: (**a**) %DNA in tail, (**b**) tail length, (**c**) tail moment, (**d**) olive tail moment, (**e**) comet image for all experimental groups. The data points are represented as mean ± SD (n = 3). The statistical difference between donates at a, p < 0.0001 and b, p < 0.001 when compared to control group.

Modulation of oxidative stress by curcumin and Cur-NE is reflected in DNA damage. Comet assay from kidney, liver, heart, spleen, and tumor tissues showed comet growth in the treatment groups. Thus, DNA damage was measured as %DNA in tail, olive tail moment, tail length and tail moment (Fig. 10a–e)^60,61^. Mice groups treated with Cur-NE revealed a significant increase in the percentage of DNA (%DNA) in tail, olive tail moment, tail length and tail moment in the kidney, liver, heart, and spleen tissues in the range (2.6–57%) compared to the control group (Fig. 10a–d). On the other hand, tumor tissues showed a nonsignificant result compared to the control group. This data were proven by the comet images in Fig. 10e. These results may be due to the small size of Cur-NE which permits it to enter mitochondria and nuclear pores and the ability of curcumin to regulate cellular redox stability by distracting mitochondrial homeostasis and improving cellular oxidative stress. Furthermore, curcumin causes ROS creation and decreases mitochondrial transmembrane potential thereby activating DNA damage/repair pathway and mitochondrial apoptosis^62^. Additionally, a nonsignificant result in tumor tissues might be due to lack of genotoxicity of the Cur dose.

### Histopathological examination

**Figure 11:**
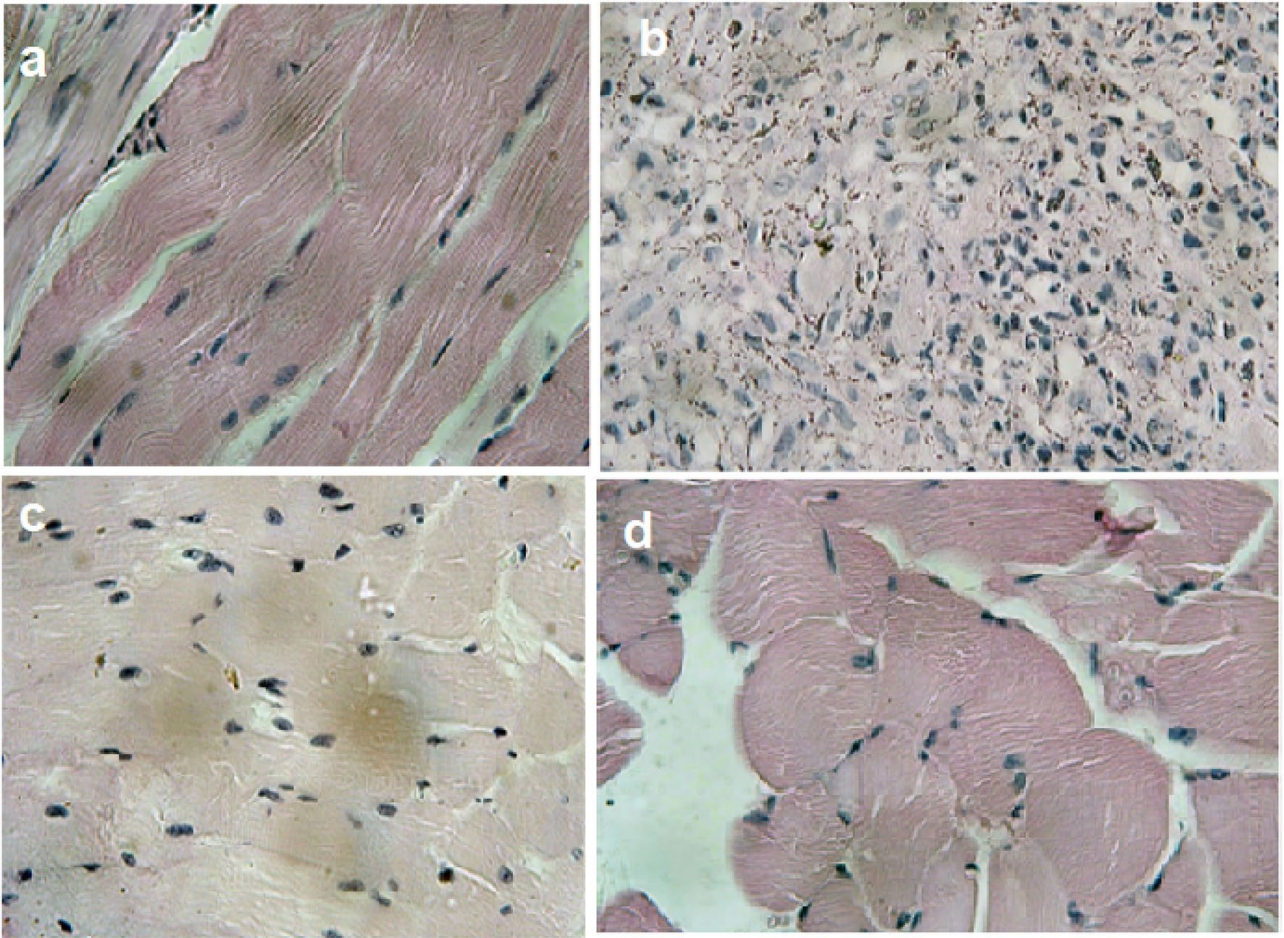
Histopathology photomicrographs for striated skeletal muscles from control mice (**a**), Ehrlich carcinoma-bearing mice (**b**), mice treated with curcumin free (**c**) and loaded in nano emulsions (**d**) × 100.

Curcumin triggers mitochondrial permeability which lead to mitochondrial swelling, loss of mitochondrial membrane potential, embarrassment of mitochondrial function, hence, initiation of cellular apoptosis in cancer cells^63^. These results are displayed in the histopathological variation in striated skeletal muscles (Fig. 11a–d). Figure 11a shows a normal striated skeletal muscle for the normal mice compared to marked pleomorphism, hyperchromatism and mitotic activity for tumor cells in the skeletal muscles in Ehrlich carcinoma-bearing mice (Fig. 11b). On the other hand, mice group treated with CR shows masses of pleomorphic and anisokaryotic tumor cells penetrating striated muscles (Fig. 11c). While the group treated with Cur-NE shows the inhibition of tumor cells embedded in the muscles, as in Fig. 11d.

### Western blot

**Figure 12:**
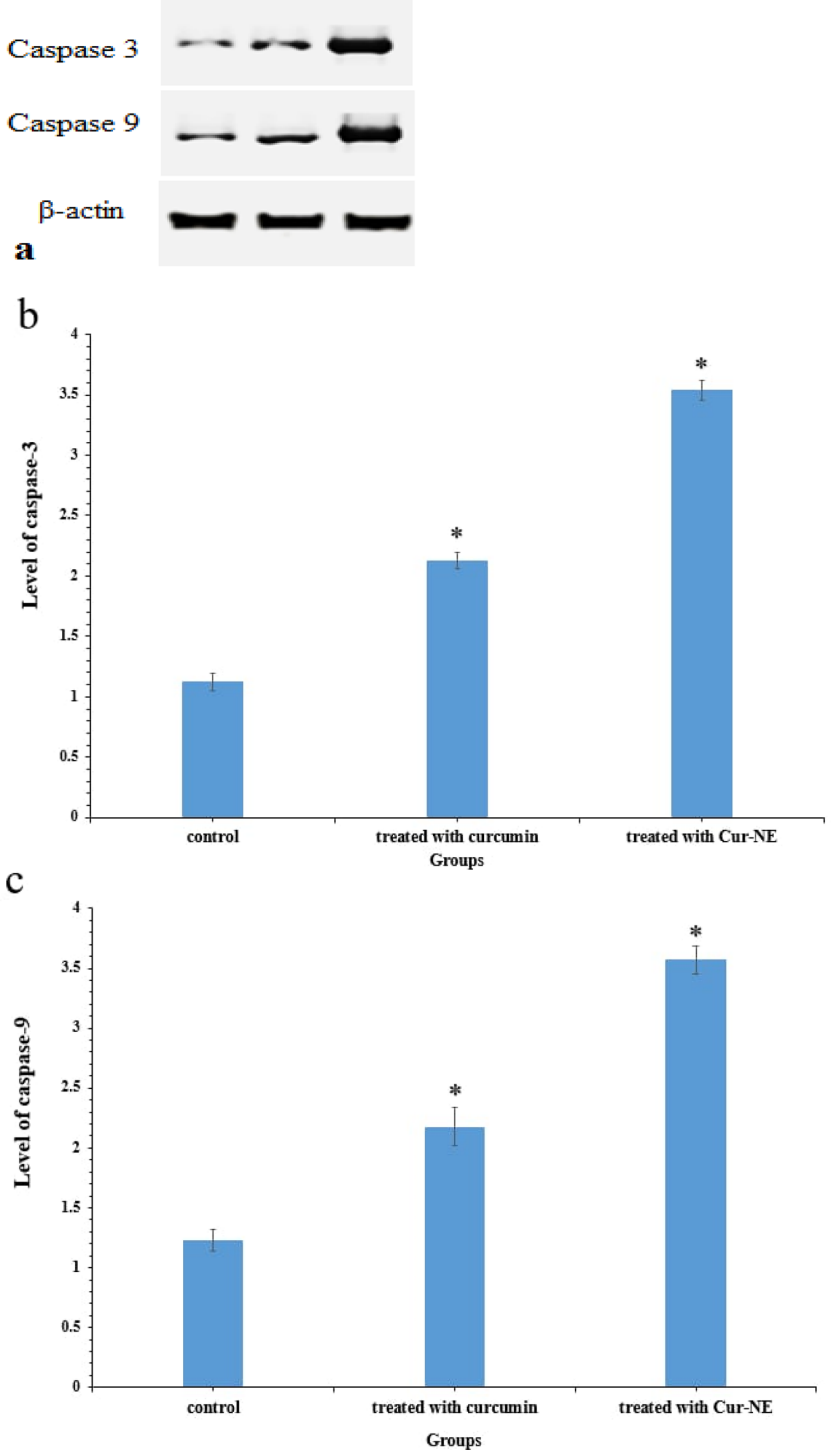
Western blotting (**a**). The level of protein expression: caspase-3 (**b**) and caspase-9 (**c**) *p ≤ 0.0001 when compared to tumor group.

Figure 12a–c illustrates the expression of apoptotic proteins in Ehrlich ascites carcinoma treated with curcumin and Cur-NE. In association to the control group, the level of caspases 3 and 9 significantly increased by around 1- and 3.5-fold, respectively. These results are consistent with the findings of other research^64,65^ that suggest curcumin can cause oxidative stress, resulting in mitochondrial malfunction and an imbalance in antioxidant defiance. Hence, apoptotic proteins are activated, resulting in cell death (Supplementary Information).

### Tumor growth

**Figure 13:**
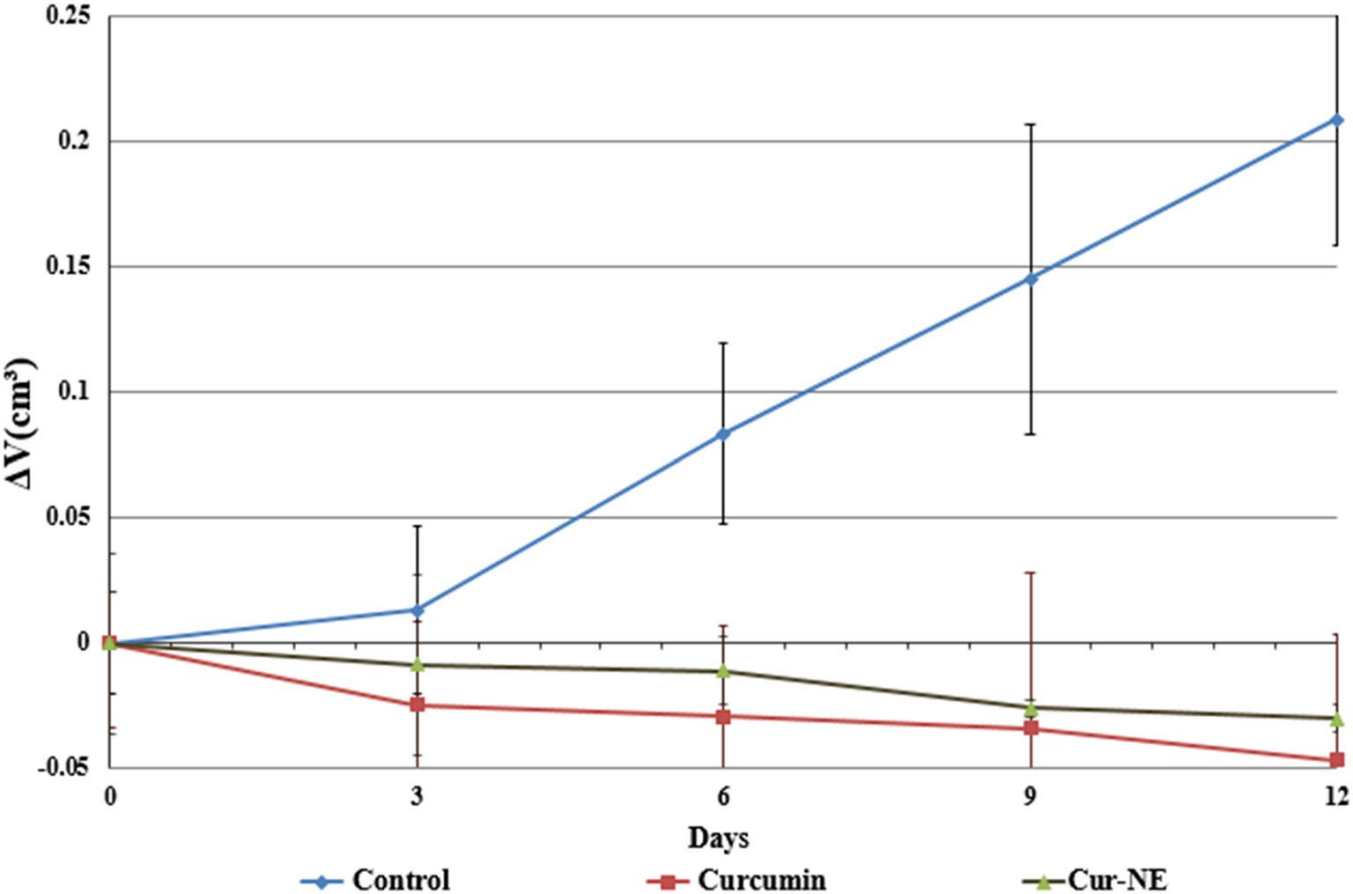
Effect of free curcumin and curcumin in nano emulsion on tumor volume (cm^3^) in curcumin (CR) and curcumin loaded in nano emulsion groups.

Curcumin and Cur-NE cause oxidative stress, DNA damage, apoptotic proteins, and histological alterations, which result in tumor shrinking and a delay in tumor growth (Fig. 13). Figure 12 shows that tumor volumes clearly rise over time in the control group. It also be noted that mice groups treated with curcumin and Cur-NE showed obstacles in tumor growth rate compared with the control one. Based on previous studies these results may be due to the ability of curcumin to generate free radical-driven apoptosis and/or necrosis of tumor cells^66^.

## Conclusion

The current Cur-NE was created and characterized using several tools. The formulation demonstrated great encapsulation efficiency and improved regulated curcumin release. Cur-NE and free curcumin were used to cure solid Ehrlich tumor implanted in Balb/c mice. According to the combined findings, Cur-NE was a successful treatment when compared to free curcumin, with little cytotoxicity for the essential organs.

### Guideline

The study is reported in accordance with Guide for the Care and Use of Laboratory Animals and Use Committee (CU-IACUC) number CU/I/F/1/21.

The current study was carried out in compliance with the ARRIVE guidelines when relevant methods were applied.

### Supplementary Information

Supplementary Information 1.

Supplementary Information 2.

Supplementary Information 3.

## Data Availability

The datasets generated during and/or analyzed during the current study are available from the corresponding author on reasonable request.
