# Supplementary figures and images for: Physico-chemical properties of curcumin nanoparticles and its efficacy against Ehrlich ascites carcinoma
Source: Sci Rep. 2023 Nov 24;13:20637. doi: 10.1038/s41598-023-47255-w (PMC10674021; doi:10.1038/s41598-023-47255-w)

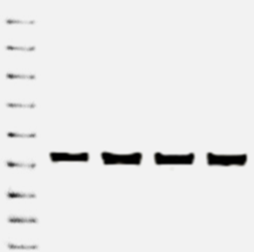

Supplement: Supplementary file 1 — Supplementary Information 1. [file 41598_2023_47255_MOESM1_ESM.tif]

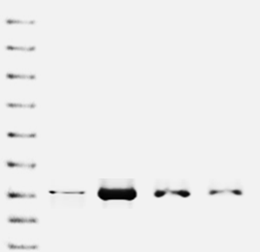

Supplement: Supplementary file 2 — Supplementary Information 2. [file 41598_2023_47255_MOESM2_ESM.tif]

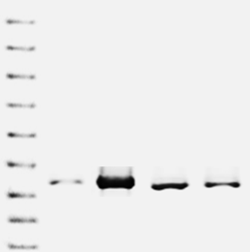

Supplement: Supplementary file 3 — Supplementary Information 3. [file 41598_2023_47255_MOESM3_ESM.tif]
